# Tumor‐Associated Macrophages: Key Players in the Non‐Small Cell Lung Cancer Tumor Microenvironment

**DOI:** 10.1002/cam4.70670

**Published:** 2025-02-10

**Authors:** Tongtong Lv, Rui Fan, Jiaqi Wu, Haolan Gong, Xiaoru Gao, Xin Liu, Yixin Gong, Bo Luo, Yanhua Zhang, Xiaochun Peng, Gai Liang

**Affiliations:** ^1^ Department of Hematology The First Affiliated Hospital of Yangtze University Jingzhou China; ^2^ Department of Pathophysiology School of Basic Medicine, Health Science Center, Yangtze University Jingzhou China; ^3^ Department of Radiation Oncology Hubei Cancer Hospital, Tongji Medical College, Huazhong University of Science and Technology Wuhan China

**Keywords:** biomarkers, drug resistance, NSCLC, TAMs, treatment

## Abstract

**Background:**

Lung cancer is among the most common and deadliest malignant tumors worldwide. It is often detected at late stages, resulting in unfavorable outcomes, with tumor cell heterogeneity and medication resistance. Tumor‐associated macrophages are among the key cells contributing to cancer progression. They are categorized into two primary phenotypes: Proinflammatory (M1) and anti‐inflammatory (M2) which are involved in the onset and progression of NSCLC. The role of common cytokines secreted by macrophages in the progression of lung cancer are described, and the effects of various substances such as RNA or protein on the differentiation and polarization of two phenotypes of macrophages are highlighted to characterize the impact of the immune state of tumors on therapeutic effect of treatments and patient prognosis. Researchers have primarily aimed to investigate innovative carriers and strategies based on macrophages to modify the tumor microenvironment.

**Objectives:**

These approaches are often integrated with other treatments, particularly immunotherapy, to enhance therapeutic efficacy.

**Methods:**

A comprehensive review was carried out by systematically synthesizing existing literature on PubMed, using the combination of the keywords “TAMs”, “NSCLC”, “Drug resistance”, and “therapy”. The available studies were screened for selection based on quality and relevance.

**Conclusions:**

TAMs promote tumor invasion, growth, and metastasis by promoting angiogenesis and EMT. In addition, they contribute to the development of drug resistance and the immunosuppressive microenvironment establishment. The immunosuppressive factors secreted by TAM can weaken the activity of immune cells, inhibit their killing effect on tumors, leading to immune suppression and hindering the effectiveness of treatment. Therefore, TAM is a key target for the development of cancer immunotherapy. Various strategies are being explored, including reducing the recruitment of TAMs and influencing their polarization to treat NSCLC. In addition, TAMs based treatment systems can achieve precise delivery of drugs or gene interfering molecules without causing side effects.

## Graphical abstract

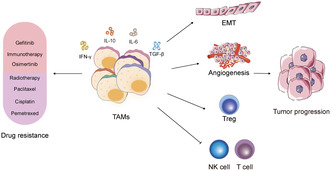

**Abbreviations**
CAFs: cancer‐associated fibroblasts
CBD: collagen‐binding domain
Ce6: chlorin e6
CSCs: cancer stem cells
DOX: doxorubicin
ECM: extracellular matrix
GM‐CSF: granulocyte‐macrophage colony‐stimulating factor
HER2/ERBB2: human epidermal growth factor receptor 2
HMSNs: hollow mesoporous silica nanoparticles
IDO1: indoleamine 2,3‐dioxygenase 1
IL‐1RA: interleukin‐1 receptor antagonist
INOS: inducible nitric oxide synthase
LID: lidocaine
LPS: lipopolysaccharide
LUAD: lung adenocarcinoma
LUSC: lung squamous cell carcinoma
MDSCs: myeloid‐derived suppressor cells
Mut: mutation
NBs: nanobubbles
NCAPD2: non‐structural maintenance of chromosome condensin I complex subunit D2
Neo: neobavaisoflavone
NIR: near infrared irradiation
NLRC3: NLR family card domain containing 3
NPs: nanoparticles
NSCLC: non‐small cell lung cancer
NTRK1: neurotrophic tyrosine kinase receptor 1
PD‐1: programmed cell death protein 1
PD‐L1: programmed death ligand 1
PEI: polyethylenimine
PG: polyaniline‐based glyco structure
PLGA: poly (lactic‐co‐glycolic acid)
PZP: pregnancy zone protein
RA‐SBRT: rapid arc‐stereotactic body radiotherapy
RNS: reactive nitrogen species
ROS: reactive oxygen species
RRM2: ribonucleotide reductase M2
RTK: receptor tyrosine kinase
SFTPA1: pulmonary surfactant protein A1
TAMs: tumor‐associated macrophages
TF: tissue factor
Th1: helper T cell 1
Th2: helper T cell 2
TKI: tyrosine kinase inhibitor
TLRs: toll‐like receptors
TMB: tumor mutation burden
TME: tumor microenvironment
TTN: titin
UMND: ultrasound‐mediated nanobubble destruction
α‐TOS: α‐tocopheryl succinate

## 1 Introduction

Lung cancer, a prevalent malignant tumor that arises from abnormal cells in lung tissue, is one of the deadliest malignancies globally [1]. Non‐small cell lung cancer (NSCLC) accounts for 85% of all lung cancer patients, and it is more common in lung adenocarcinoma (LUAD) and lung squamous cell carcinoma (LUSC). Several treatments can be used for cancer treatment. Conservative surgical intervention, radiotherapy and chemotherapy, targeted therapy, immunotherapy or combined therapy play an important role in stabilizing the disease and reducing recurrence and pain [2, 3]. Despite the availability of various treatment options, the prognosis of NSCLC is poor [1], mainly due to late diagnosis. Furthermore, tumor cell heterogeneity and drug resistance influence the therapeutic impact of NSCLC interventions, contributing to treatment instability and unpredictability [4, 5].

The tumor microenvironment (TME), which is necessary for tumor survival, comprises tumor‐associated macrophages (TAMs), cancer‐associated fibroblasts (CAFs), immune cells, stromal cells, endothelial cell, neuron cells, and myeloid‐derived suppressor cells (MDSCs), and the extracellular matrix (ECM). These cells communicate with each other through various cytokines, and cooperate or compete, thus influencing tumor growth, metastasis, and invasion [6]. The TME is also closely linked to drug resistance, and a more in‐depth exploration into its impact on therapeutic responses is essential for developing new treatment strategies and drugs [7, 8]. TAMs play an important role in it.

In this review, we describe the role of some cytokines secreted by TAMs in NSCLC, such as IL‐6 and TGF‐β. In the process of NSCLC progression and drug resistance, M2 macrophages play a major role in promoting tumor. We also summarize the biomarkers for predicting the prognosis and therapeutic effect of NSCLC, and find that these biomarkers are related to the infiltration of immune cells, including M1 macrophages. In addition, the development and application of some therapeutic systems activate the immune response and change the ratio of M1/M2 macrophages in the process of tumor progression.

## 2 Biological Function OF TAMs

**FIGURE 1:**
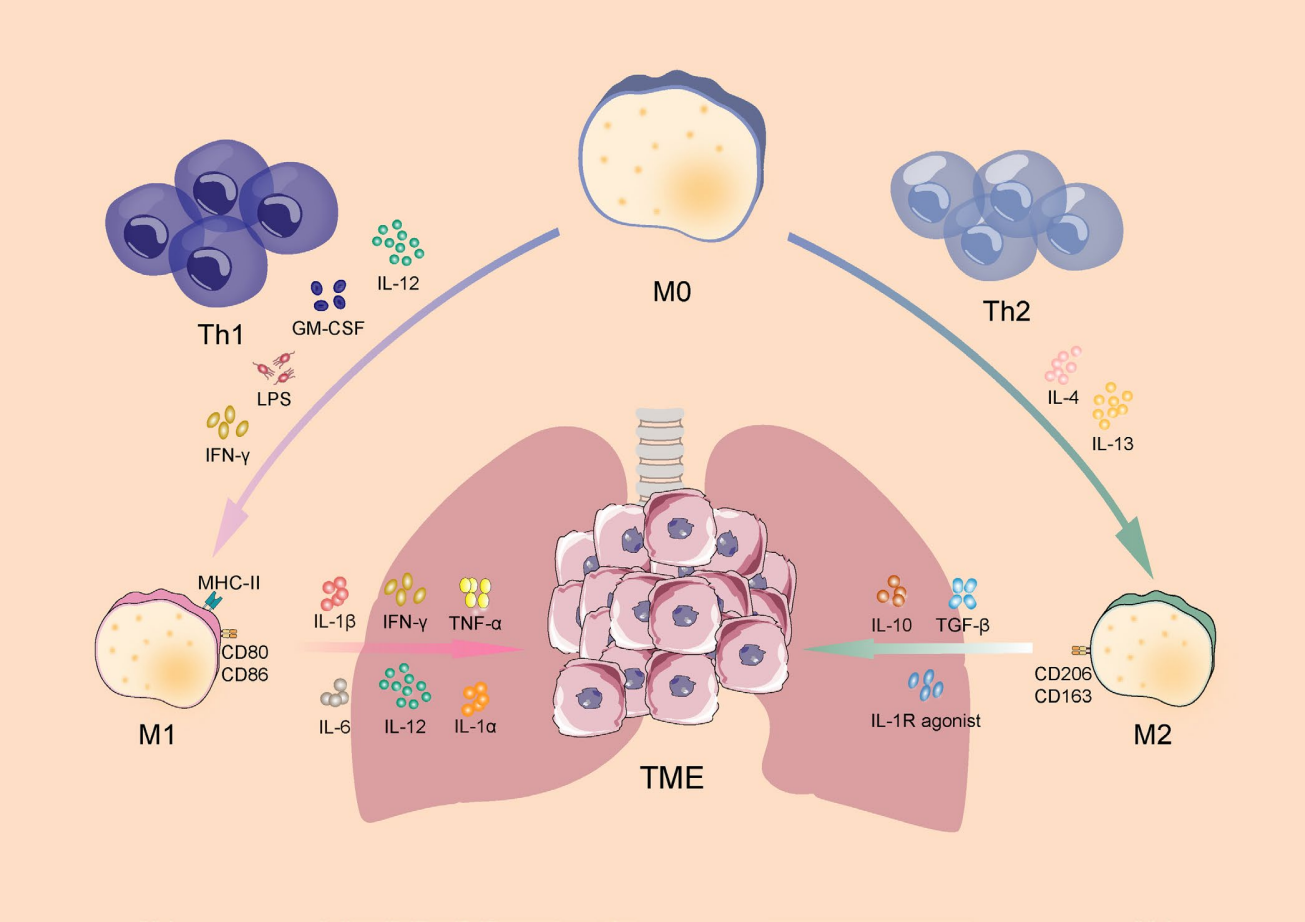
TAMs polarization. TAMs, stimulated by various cytokines, are polarized into M1 and M2 phenotypes in TME.

Macrophages, a crucial immune system component, are found in almost all tissues. Besides their diverse functionality, macrophages exhibit significant phenotypic heterogeneity [9]. Macrophages are divided into classically activated M1 macrophages and selectively activated M2 macrophages based on the characteristics of their cell surface marker molecules, cytokine secretion, and metabolism‐related genes. These two main phenotypes of macrophages affect tumor immune microenvironment. Abnormal activation of the M2 macrophages enhances the malignant transformation of tumor cells [10, 11, 12] (Figure 1).

M1 macrophages are commonly found in inflammatory environments, where they promote inflammatory responses against invading pathogens and tumor cells while participating in the helper T cell 1 (Th1) response to pathogens. It has been established that Th1 cytokines such as IFN‐γ, IL‐12, and IL‐18, as well as activated toll‐like receptors (TLRs), such as lipopolysaccharide (LPS) and granulocyte‐macrophage colony‐stimulating factor (GM‐CSF) [13, 14, 15], polarize macrophages towards the M1 phenotype, thus regulating tumor growth and metastasis and inducing Th1 response. Additionally, M1 macrophages secrete cytokines such as IL‐1α/β, IL‐6, IL‐12, TNF‐α and IFN‐γ, which contribute to the immune response. Furthermore, these cells express reactive oxygen/nitrogen species (ROS/RNS) and produce inducible nitric oxide synthase (iNOS) [16]. Moreover, the high expression of MHC class II, CD80 and CD86 on the cell surface increases the ability of macrophages to recognize pathogens more effectively, and promotes the phagocytosis of M1 macrophages and the ability to kill target cells, thus better responding to the immune process [17]. Therefore, M1 macrophages are crucial for controlling efficient immunological reactions to microbial activity, tumor suppression, immune system activation, and host resistance to infection.

Research has demonstrated that M2 macrophages are primarily activated by helper T cell 2 (Th2) cytokines. The secretion of IL‐4 and IL‐13 enhances the phenotypic polarization of M2 [15]. M2 macrophages are often anti‐inflammatory in nature, secreting low levels of IL‐12 and high levels of IL‐10 [18]. They inhibit immune response, promote angiogenesis, and alleviate pain, enhance wound healing, and bone regeneration by increasing the secretion of IL‐10, TGF‐β, and interleukin‐1 receptor antagonist (IL‐1RA) [19, 20, 21, 22]. M2 macrophages have poor antigen‐presenting ability, and usually express the surface markers CD206 and CD163. Besides IL‐10 and TGF‐β, Arg1 contributes to immunosuppression [23].

## 3 TAMs Affect the Development of NSCLC

**FIGURE 2:**
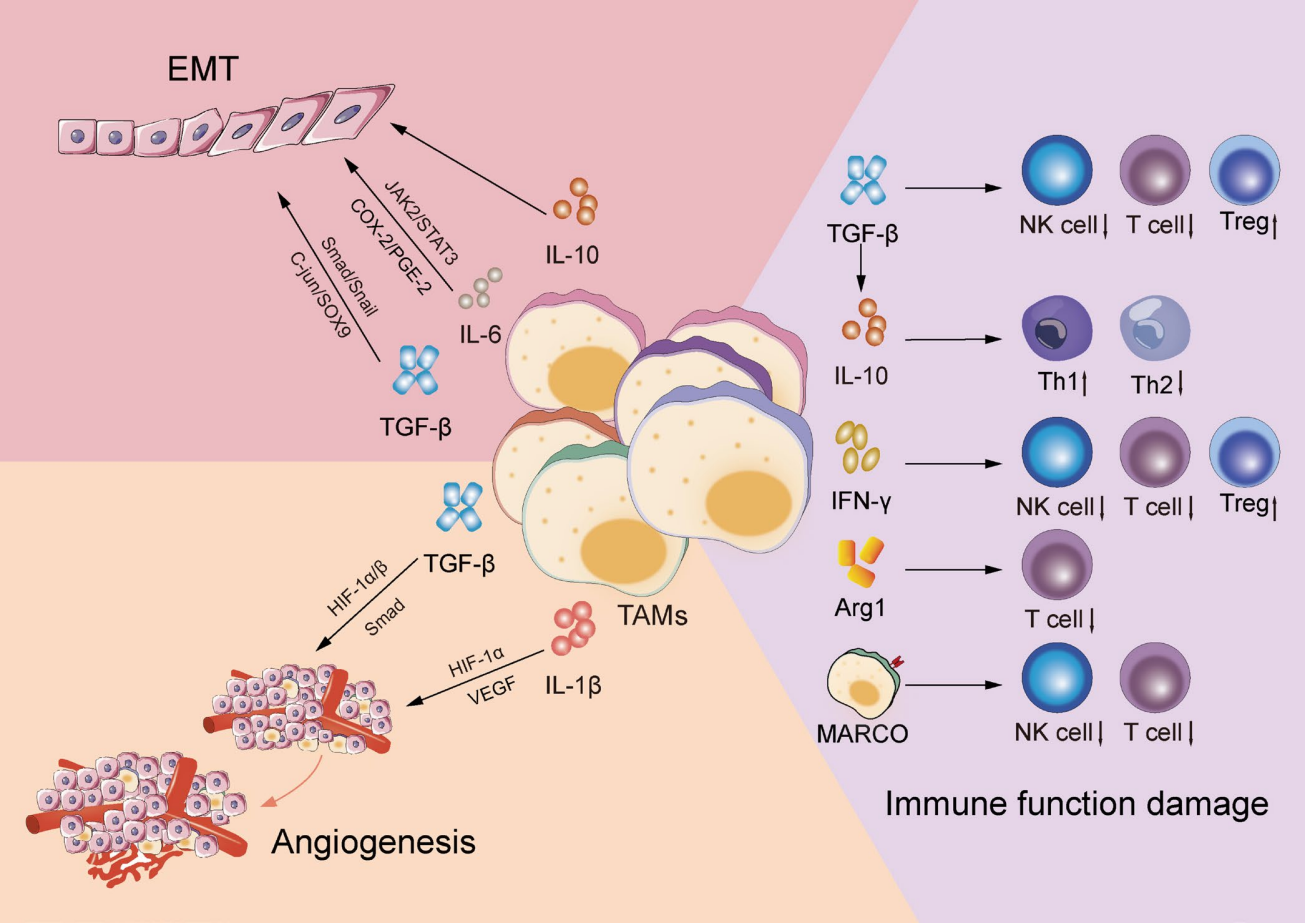
Pathway of TAMs in affecting NSCLC progression. TGF‐β and other TAMs‐related substances activate some mechanisms, such as Smad and AKT to promote EMT and angiogenesis, and inhibit immune cell function.

It has been established that TMs are involved in practically every tumor progression step, and a positive feedback exists between tumor cells and TAMs in NSCLC. It is through kinds of signaling molecules that TAMs directly influence tumor cell development, invasion, and metastasis and help create an environment conducive for tumor cell growth, such as TGF‐β, IL‐10. Table 1 reveals the influence of these cytokines in TME of NSCLC, including creating an environment that favors the development and migration of NSCLC and inhibiting immune response. The activated pathway is presented in Figure 2.

**TABLE 1:** TAMs‐related cytokines affect the development of lung cancer.

Feature	Cytokine	Mechanism	Reference
EMT	TGF‐β	Smad signaling activation and Snail upregulation	[25, 26]
		TGF‐β/SOX9 axis	[27]
	IL‐6	COX‐2/PGE‐2	[28]
		JAK/STAT	[29]
	IL‐10	TLR4/IL‐10	[31]
Angiogenesis	IL‐1β	VEGF‐A release and HIF‐1α expression	[33]
	TGF‐β	HIF‐1α/β and Smad3/4 signaling pathways	[34]
Immune function damage	TGF‐β	Inhibiting NK and T cells; promoting the Tregs	[35, 36]
	IL‐10	Activating Th2 cells and suppressing Th1 cells	[37]
	IFN‐γ	Inhibiting T and NK cells; promoting the Tregs	[38]
	MARCO	Inhibiting the NK and T cells	[39]
	Arg1	Inhibiting the T cells	[40, 41]

### 3.1 EMT Occurrence

EMT endows the tumor with malignant biological characteristics, accelerating tumor progression. During EMT, epithelial cells lose their cell adhesion molecules (such as E‐cadherin) and strongly regulate the expression of mesenchymal molecules (such as Waveform protein and β‐catenin) and transcription factors (e.g., Snail) [24]. TAMs interact with epithelial cells by secreting various cytokines such as TGF‐β and IL‐6, thus triggering or promoting EMT.

In NSCLC, the TAM‐released TGF‐β promotes EMT occurrence via SMAD cascade signaling activation (Smad 2/3 and Smad 1/5/8) and Snail upregulation, thus triggering EMT [25, 26]. Additionally, TAMs can enhance SOX9 expression via the c‐Jun pathway or activate the β‐linked protein pathway to induce EMT in NSCLC [27]. Moreover, some TAM‐produced interleukin factors exert essential effects on tumor EMT. Co‐cultivation of THP‐1‐derived macrophages with A549 and H1299 lung cancer cells in vitro revealed that the IL‐6 system greatly aided the EMT process in NSCLC cancer cells via the IL‐6‐dependent COX‐2/PGE‐2 signaling pathway regulation [28]. Additionally, IL‐6 activated the STAT3 tyrosine phosphorylation signaling pathway, promoted the M2 polarization of TAMs [29], and indirectly stimulated EMT in NSCLC cancer cells [30]. Furthermore, in an LLC cell model, IL‐10 enhanced tumor cell resistance to apoptosis and activated the TLR4/IL‐10 cascade reaction, thus promoting tumor cell EMT [31].

### 3.2 Angiogenesis

Angiogenesis is a critical process contributing significantly to tumor growth, spread, and progression. New blood vessel formation substantially promotes tumor growth, these blood vessels supply tumor cells with the required oxygen and nutrients and efficiently eliminate metabolites. Multiple factors, including TAMs, have been established to regulate neovascularization. Notably, VEGF, also known as the vascular permeability factor, is a highly specific homodimer glycoprotein that binds to heparin and enhances blood vessel permeability, which causes damage to the ECM. Additionally, VEGF promotes EC migration and growth in the arteries, facilitating the formation of new blood vessel [32]. Studies have shown that TAMs induce the release of HIF‐1α and VEGF‐A in tumor tissues via increasing IL‐1β production [33]. Additionally, TAM‐secreted TGF‐β promoted VEGF‐A expression via HIF‐1α/β and Smad3/4 dependent mechanisms in rat macrophages [34].

### 3.3 Immune Function Damage

TGF‐β directly regulates the expression of cytolysis‐related genes, including granzyme A, granzyme B, and IFN‐γ, and enhances the development of Tregs, which in turn inhibits the activity of highly effective T cells, either directly or by releasing immune‐suppressing factors [35]. Furthermore, TGF‐β inhibits NK cell cytotoxicity via cell membranes, lowering the efficacy of NK cells in tumor immunosurveillance [36]. These mechanisms limit the capacity of immune system to eradicate lung cancer cells. In addition, IL‐10 overexpression in tumor cells was induced by TGF‐β, which subsequently activated Th2 cells while suppressing Th1 immune activity. This shift disrupted the balance between Th1 and Th2 cells, leading to macrophage polarization towards the M2 phenotype, thereby inhibiting the immune‐mediated elimination of tumor cells [37]. Low IFN‐γ concentrations in stage IA NSCLC enhanced cancer cell activity via Indoleamine 2,3‐dioxygenase 1 (IDO1) upregulation, IDO1 activity regulates tryptophan consumption and is linked to cellular circuits specifically initiated by IDO1+ cells, contributing to Treg accumulation and severe impairment of T cell and NK cell function. Moreover, in such a setting, TAMs are more likely to be polarized into M2 phenotypes, thereby excerbate tumor progression [38]. Moreover, lung cancer M2‐polarized macrophages secrete the macrophage receptor with collagenous structure (MARCO), which inhibits the activation and proliferation of cytotoxic T cells and NK cells, along with their cytokine production. This suppression ultimately reduces the ability of these immune cells to eradicate tumors [39]. Furthermore, TAMs can express Arg1, an enzyme that depletes l‐arginine in the surrounding environment. This depletion inhibits T cell expression and proliferation, thereby disrupting T cell metabolism and cytotoxicity [40, 41]. TAM‐secreted IL‐10, TNF‐α, and IFN‐γ can induce B7‐H4 production in lung cancer cell surfaces, B7‐H4 can cause T‐cell apoptosis and prevent CTL‐mediated cytolysis. These processes help lung cancer cells to evade recognition and clearance by the immune system [42, 43].

## 4 TAMs Promote Drug Resistance in NSCLC

**FIGURE 3:**
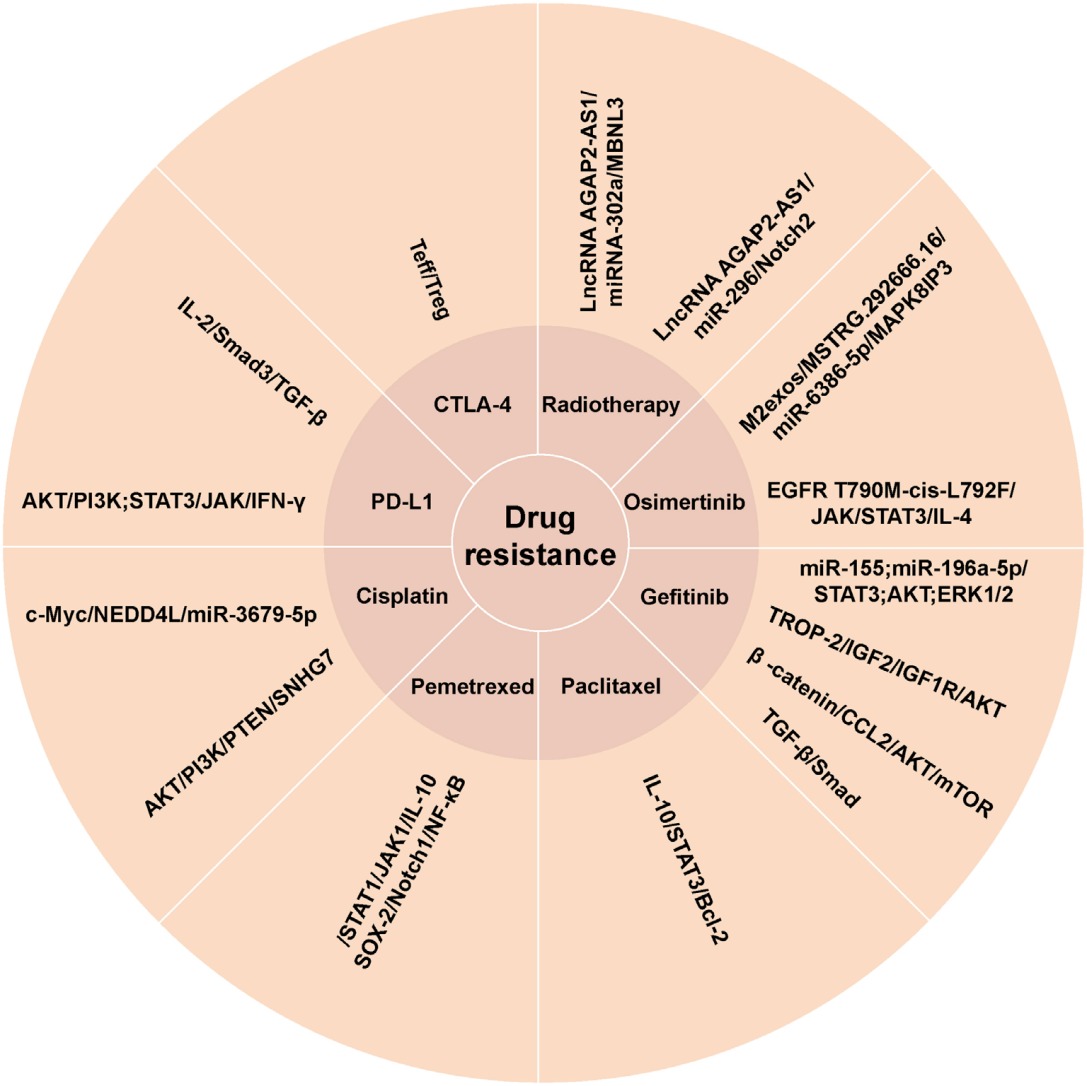
TAMs influence drug resistance in NSCLC. TAMs‐related substances such as IL‐10; TGF‐β; IFN‐γ and exosomes released by M2‐type TAMs affect chemotherapy (paclitaxel, cisplatin, etc.), radiotherapy, target therapy (gefitinib, osimertinib, etc.) and immunotherapy (PD‐L1 and CTLA‐4 inhibitors) through some pathways.

The mechanism of drug resistance is complex. Apart from gene mutations, some signaling pathways in NSCLC cells are activated by soluble factors released by TAMs and extracellular vesicles to trigger therapeutic resistance (Figure 3). These findings offer a fresh perspective on the mechanisms underlying drug resistance in chemoradiotherapy and targeted therapy. They also identify potential targets for the development of new therapeutic strategies aimed at overcoming these resistance mechanisms.

### 4.1 Chemotherapy

A549 cells can downregulate NEDD4L expression by absorbing miR‐3679‐5p produced by M2 macrophages, which alleviates NEDD4L‐mediated c‐Myc ubiquitination. Therefore, A549 cells exhibit high expression of the c‐Myc protein, which makes tumor cells more resistant to cisplatin [44]. Exosome SNHG7 induces M2 TAMs polarization by down‐regulating PTEN and activating the PI3K/AKT pathway. It also enhances cisplatin resistance in LUAD cells [45]. The P2X7 expression in TAMs was reported by Qin et al. who found that it can stimulate lung tumor growth and induced resistance of CLL cells to PD‐1 and cisplatin [46]. It has been reported that the resistance to anti‐tumor therapy is influenced by the interaction between TAMs and cancer stem cells (CSCs). Notably, the CSCs release chemokines which regulate the recruitment of macrophages. The CSCs stemness in NSCLC contributes to the occurrence of pemetrexed resistance. TAM produce IL‐10 which improves the viability of cancer cells in NSCLC by stimulating the JAK1/STAT1/NF‐κB/Notch1 signaling pathway and promoting the expression of SOX‐2 and other genes linked to cancer cells [47]. M2 TAMs secrete large amounts of IL‐10 in the TME, which stimulate JAK/STAT signaling in the tumor, lowers paclitaxel's effectiveness via the IL‐10/STAT3/Bcl‐2 cascade, and enhances paclitaxel resistance [48].

### 4.2 Targeted Therapy

Research has demonstrated that exosomes generated by M2 macrophages contain miR‐155 and miR‐196a‐5p, which inhibits the efficacy of gefitinib against lung cancer cells by activating the AKT, ERK1/2, and STAT3 signaling pathways, thereby inducing therapeutic resistance [49]. Sun et al. found that trophoblast cell surface antigen‐2 promotes the IGF2‐IGF1R‐AKT axis after binding to insulin‐like growth factor 2 receptors to promote gefitinib resistance in NSCLC and remodels the TME [50]. Xiao et al. showed that gefitinib‐resistant cell lines can release a large amount of CCL2 and attract M2 macrophages by reducing the expression of β‐catenin. This process also forms a part of gefitinib resistance mechanism by activating AKT/mTOR pathway [51]. Evidence indicates that TAMs enhance primary resistance to gefitinib via the TGF‐β/Smad signaling pathway [52]. Osimertinib is a first‐line or salvage treatment for EGFR mutation NSCLC, its resistance is common. M2exos drive the occurrence of osimertinib resistance in NSCLC by modulating the MSTRG.292666.16/miR‐6386‐5p/MAPK8IP3 axis [53]. Sun et al. showed that EGFR T790M‐cis‐L792F variant stimulated the JAK/STAT3 pathway, which in turn promoted the specific binding of p‐STAT3 (Tyr705) to the IL‐4 promoter. This leads to increased IL‐4 production and expression, causing M2 macrophages to become more polarized and acquire osimertinib resistance in NSCLC cells [54].

### 4.3 Radiotherapy

The hypoxic TME is a natural strategy for reducing radiation response in malignant tumors. By inducing apoptosis and decreasing radioresistance, the insertion of the lncRNA AGAP2‐AS1 affects the miRNA302a/MBNL3 axis and decreases NSCLC's resistance to radiochemotherapy [55]. Lung cancer cells uptake the lncRNA AGAP2‐AS1, which is produced by exosomes from M2 macrophages. This particular inhibition of miR‐296 raises the expression of the downstream target gene NOTCH2. Data indicates that activating this signaling pathway boosts resilience of lung cancer cells to radiation therapy while also decreasing the capacity of NK cells to eradicate lung cancer cells [56].

### 4.4 Immunotherapy

Immunotherapy is one of the current methods for treating tumors and aims to improve the capacity of own immune cells to kill tumor cells. The immune surveillance receptor PD‐1/PD‐L1 signaling pathway has been reported to play a role in the regulation of TME, allowing lung cancer cells to evade the anti‐cancer immune responses [57, 58]. In vitro experiments revealed that M2 macrophages promoted PD‐L1 expression in tumor cells via TGF‐β secretion, increasing the chances of immune escape from the tumor cells [36]. TGF‐β inhibits the production of IL‐2 through the intrinsic Smad3 signaling pathway of T cells in the TME. This subsequently inhibits the activation of T cells in advanced NSCLC. This reduces the effectiveness of PD‐1/PD‐L1 drugs in blocking inhibitory signals for T cell activation, allowing tumor cells to evade T cell immune responses and ultimately leading to immune tolerance [59]. The study also found that the combination of anti‐PD‐L1 and anti‐TGF‐β therapy boosted T cell infiltration in patients with NSCLC who were resistant to immune checkpoint drugs. This implies that by preventing T cell accumulation in the TME, TGF‐β reduces the efficacy of anti‐PD‐L1 treatment [60]. TAM‐secreted IFN‐γ stimulates the PI3K/AKT and JAK/STAT3 signaling pathways in A549 cells, increasing PD‐L1 expression and migration in tumor cells [38, 58, 61]. Generally, IFN‐γ suppresses T cells and upregulates PD‐L1, resulting in immunosuppression around the tumor. Moreover, TAMs interact with Teff and Treg cells to induce adaptive immunological tolerance to CTLA‐4 inhibitors [43].

## 5 TAMs and Biomarkers of NSCLC

The expression of various proteins and genes associated with TAMs influences the treatment response and prognosis of patients. In addition, they affect the infiltration and phenotype of immune cells in tumors, by altering the enrichment of TAMs and their subsets (Table 2).

**TABLE 2:** Functions of biomarkers related to TAMs.

Type	Name	Function related to TAMs	Reference
High expression biomarkers	NCAPD2	Affecting the infiltration of M1 macrophages	[62]
	ADAR1	Increasing M2 macrophages; decreasing M1 macrophages	[63]
	RRM2	Affecting the M1/M2 macrophages	[64]
	RAB40C	Decreasing M1 macrophages	[65]
	CDK1	Poor response to immunotherapy; poor clinical outcomes	[66]
	FGFR4	Independent predictor of ICI efficacy	[67]
	LIPG	Decreasing drug resistance	[68]
	SIRPG	Good response of PD‐1 blocking	[69]
Low expression biomarkers	PZP	Increasing M1 macrophages; decreasing M2 macrophages	[70]
	JAK1	Gene marker of M1 macrophages	[71]
	SFTPA1	Increasing M1 macrophages	[72]
	GPRC5A	Correlating with the distribution of tumor PD‐L1	[73]
	NLRC3	Improving efficacy of immunotherapy	[74]
Mutation biomarkers	EPHA5	Increasing M1 macrophages	[75, 76]
	TTN	Increasing M1 macrophages	[77]
	NTRK1	Improving the therapeutic effect of ICI	[78]

### 5.1 High Expression Biomarkers in NSCLC

Non‐structural maintenance of chromosome condensin I complex subunit D2 (NCAPD2) has been reported to regulate cell mitosis. Compared with normal samples, the expression level of NCAPD2 in tumor samples was reported to be significantly upregulated. Immuno‐infiltration analysis showed that the NCAPD2 affected the infiltration of B cells, CD4+ T cells and M1 macrophages. Silencing NCAPD2 suppressed the proliferation, migration, invasion, EMT, and cell cycle process of LUAD cells [62]. The influence of NCAPD2 on the lung cancer is mediated by its capacity to regulate the cell cycle. Further studies are needed to identify the detailed mechanism by which NCAPD2 modulates lung cancer. ADAR1 (RNA‐editing enzyme can lead to the creation of missense mutations in coding sequences) is highly expressed in lung cancer tissues, which also increases the expression of tumor cells such as M0 and M2 macrophages, but decreases the levels of CD4+ T cells in the effector immune cells. In addition, the ADAR1 expression in patients with lymph node metastasis is negatively correlated with the content of M1 macrophages [63]. Therefore, this RNA editing may be a potential strategy to treat lung cancer patients. Ribonucleotide reductase M2 (RRM2) which regulates ferroptosis can also influence the progression of LUAD and immune cells infiltration in tumor. RRM2 inhibition increased the number of M1/M2 macrophages. Treatment with ferrostatin‐1, a ferroptosis inhibitor, effectively rebalanced macrophage polarization mediated by RRM2 inhibition [64]. Ferroptosis is a relatively new regulatory form of cell death. Its role in lung cancer needs to be further clarified. The expression of RAB40C has been shown to be increased in LUSC. In contrast, CD4+ T cells, γδT cells and M1 macrophages were decreased, while Tregs and natural killer cells were activated. RAB40C, a member of RAS oncogene family, activates the innate immune response but negative effects on adaptive immune response and affects the immune infiltration, thereby affecting the immune response [65]. In LUAD, CDK1 was significantly up‐regulated at mRNA and protein levels. Patients with high CDK1 expression exhibited poor response to immunotherapy, but good sensitivity to chemotherapy and targeted drugs. Moreover, it is accompanied by poor clinical outcomes and increased number of immune cells (including T cells and M1 macrophages) [66]. The varying reactivity and expression of CDK1 in response to different treatments facilitate immune cell infiltration but also indicate a poor prognosis, highlighting the complexity of its functional roles. The change of FGFR4 is related to higher level of tumor mutation burden (TMB) in tumor stroma, more CD8+ T cells and higher M1/M2 ratio of TAMs in tumor center and stroma. Therefore, changes in FGFR4 may be used as an independent predictor of ICI efficacy in NSCLC [67]. The expression of LIPG in LUAD tissue is increased, which correlates with poor prognosis. The expression level of LIPG is related to CD4+ T cells and M1 macrophages. Patients with high LIPG expression exhibit good response to various anti‐tumor drugs, and its expression decreases drug resistance [68]. Cancers exhibiting high levels of SIRPG tend to have increased abundances of M1 macrophages and cytotoxic lymphocytes, along with elevated levels of immunoregulatory factors. In NSCLC and melanoma, high SIRPG expression is associated with a favorable response to PD‐1 blockade [69].

### 5.2 Low Expression Biomarkers in NSCLC

The expression of pregnancy zone protein (PZP) in LUAD tissue was found to decrease significantly, leading to poor prognosis prediction. The expression of PZP increases of the number of CD4+ T cells, M1 macrophages, and decreases the number of M2 macrophages [70]. The significantly decreased expression of JAK1 in patients with LUAD correlates with poor prognosis. JAK1 is considered a gene marker of M1 macrophage, suggesting that it modulates immune infiltration [71]. Pulmonary surfactant protein A1 (SFTPA1) is a member of the C‐type lectin subfamily. SFTPA1 is involved in maintaining the homeostasis of lung tissue, and its functional damage is related to the development of lung cancer. Immune infiltration analysis revealed that elevated SFTPA1 mRNA was associated with good prognosis, increased number of M1 macrophages, CD8+ T cells and CD4+ T cells [72]. The expression of GPRC5A was negatively correlated with the infiltration of TME CD8+ T cells, CD4+ T cells and M1 macrophages. In addition, the distribution of tumor PD‐L1 expression was correlated with the expression of GPRC5A, suggesting that it may have immunotherapy effects [73]. The levels of the NLR family card domain containing 3 (NLRC3) in LUAD was reduced, especially in advanced tumors, correlated with poor prognosis. NLRC3 can promote the polarization of M1 macrophages, increasing NLRC3 expression and improve efficacy of immunotherapy [74].

### 5.3 Mutation Biomarkers in NSCLC

It was found that CD8+ T cells and M1 macrophages in patients with EPHA5 mutation (Mut) were increased significantly. A previous study found that the survival time of patients with EPHA5 mutation LUAD who received immunotherapy was significantly prolonged [75]. EPHA5 is a membrane protein that regulates cell signal transmission. It also serves as a drug target for the treatment of LUAD and a biomarker [76]. Titin (TTN) mutation increases the enrichment of macrophage M1 and is related to TMB and positively related to the prognosis of LUSC [77]. Experiments have shown that knock‐out or entrectinib (a novel oral tyrosine kinase inhibitor with central nervous system activity) therapy can improve the therapeutic effect of ICI by inhibiting the neurotrophic tyrosine kinase receptor 1 (NTRK1) pathway. Inhibition of the NTRK1 signal in tumor cells upregulates the expression of the complement C3, thereby enhancing the recruitment of T cells and stimulating the polarization of M1‐like macrophages in tumors [78].

## 6 TAMs‐Related NSCLC Treatment Strategies

Researchers have increased research efforts to develop new treatments for LUAD. For instance, cell‐based biological systems [79] and nano‐systems based on various polymer materials [80] are continuously being studied as potential drug coatings and drug delivery systems.

### 6.1 Single Treatment Strategies

The SIRPαFc fusion protein can be used as bait receptor to block the immunosuppressive signal, but it is associated with detrimental effects on cells. Using TKKTLRT, a collagen‐binding domain (CBD), the TKTLRT‐SIRPαFc conjugate was synthesized which exhibited good targeting and avoided unwanted contact with normal cells. The TKLRT‐SIRPαFC Conjugate has better anti‐tumor effect, increasing the number of M1 macrophages and maintaining stable performance [81]. Protamine‐NCs loaded with poly(I:C) + R848 showed good biocompatibility on primary macrophages. It was increased CD86 expression and reduced the expression of CD206 and Arg1 in treated mice [82]. ROS, NO and autocatalytic cascade reaction jointly activate M1 polarization. To promote the production of NO and ROS, chiral ruthenium nanozyme (D/L‐ arginine@Ru) was induced and synthesized with arginine, the precursor of NO to enhance the polarization of macrophage M1 and reverse tumor immunosuppression [83]. A liposomal nanoparticle loaded with cyclic dinucleotide and aerosolized (AeroNP‐CDN) was previously used for CDN targeting. The AeroNP‐CDN can relieve immunosuppressive TME by reprogramming TAMs into M1 phenotype and increasing tumor‐infiltrating CD8+ T cells [80]. Rapid arc‐stereotactic body radiotherapy (RA‐SBRT) effectively targets lesions while minimizing damage to normal tissues. This approach can facilitate the reprogramming of M1 macrophages and enhance the tumor immune response [84]. Neobavaisoflavone (Neo), an isoflavone derived from *Psoralea corylifolia* L., is delivered through a novel nanoemulsion (nano‐Neo) that improves its solubility and bioavailability. Nano‐Neo promotes M1 macrophage reprogramming, enhances the tumor immune microenvironment, effectively inhibits tumor growth, and exhibits minimal side effects [85]. Additionally, an ultrasound‐microbubble‐mediated gene transfer system (USMB)‐shMincle significantly boosts M1 macrophage activity in xenotransplantation tumors and suppresses tumor progression by blocking the mincle/Syk/NF‐κB signaling pathway [86]. A siRNA that silences STAT3 in tumor cells and M2 macrophages were found to be efficacy, resulting in a significant decrease in STAT3 expression and the transformation of macrophages to M1 phenotype [87].

### 6.2 Combined Treatment Strategies

The use of M1‐Exos can inhibit the growth of tumors. The vector of M1‐Exos not only kill tumor cells, but show good potential to wrap cisplatin thereby enhancing its anti‐lung cancer effect [79]. The Bi‐based mesoporous upconversion nanophosphor (UCNP) loaded with doxorubicin (UCNP‐DOX) promotes the death of immunogenic tumor cells and transforms the repolarization of the TAMs into anti‐tumor M1‐like type, which improves the tumor‐specific anti‐tumor immune effect of X‐ray radiotherapy [88]. Hollow mesoporous silica nanoparticles (HMSNs) were prepared for loading DOX, IL‐12 and α‐tocopheryl succinate (α‐TOS). N‐IL‐12/DOX/α‐TOS can achieve local release of contents in the TME by generating biological signals. In addition, N‐IL‐12/DOX/α‐TOS promotes the polarization of TAMs into M1 phenotype, which thereby improving the anti‐tumor effects, suggesting it has good combined therapy and local therapy [89]. M1‐like macrophages containing celastrol nanoparticles (NPs) were used as a combined therapy system (NP@M1) to treat peritoneal carcinomatosis of lung cancer. M1‐like macrophages not only serve as drug carriers of celastrol, but also as biotherapeutics. Conversely, the celastrol nanoparticles improve the anti‐cancer polarization of M1‐like macrophages, and exocytosis NPs can also kill tumor cells [90]. Photodynamic therapy is a new therapeutic strategy. The macrophage delivering photosensitizer chlorin e6 (Ce6) system has dual functions. For instance, the TAMs loaded with Ce6 achieve targeted delivery of Ce6 and kill lung cancer cells. Near infrared irradiation (NIR) can enhance the phagocytic function of macrophages and induce their polarization into the M1 phenotype. These dual effects synergistically inhibit the proliferation of lung cancer cells [91]. A tumor‐targeted HC/pIL‐12/polyMET micelle complex is used for co‐loading and co‐delivery of cisplatin and plasmid encoding interleukin‐12 gene (pIL‐12). The combination of cisplatin and pIL‐12 promotes the activation of immune cells, increases the level of IFN‐γ, and induces M1 polarization of TAMs, showing a synergistic therapeutic effect [92]. BMS‐202/DOX@BP complex is a drug delivery system with DOX and BMS‐202 (blocking PD‐L1) loaded on nanoparticles, which can rapidly crack and release drugs under controlled specific conditions. Low‐dose DOX and BMS‐202 delivered by BMS‐202/DOX@BP induce M1 macrophages to produce synergistic anti‐tumor response [93]. A lactoferrin‐modified liposome co‐delivery system is used for the combined treatment of Osimertinib and Panobinostat, which is an epigenetic regulator of histone acetylation. Through co‐delivery of liposomes, TAMs can be effectively reprogrammed into the M1 phenotype. This approach not only counteracts EMT‐related drug resistance in tumor cells but also suppresses malignant behaviors, including glycolysis, lactic acid production, and angiogenesis in the TME [94]. A previous study adopted the deformable lipome system (D‐lipo) to achieve combined administration of vorinostat and simvastatin to reshape TME. D‐Lipo inhibited the growth of xenograft tumors, by increasing the number of M1 macrophages and CD8+ T cells, and by anti‐angiogenesis and improving the immune environment [95]. Endoplasmic reticulum‐modified liposomes (MSLs) and lidocaine hydrochloride (LID) preloaded with the STAT3 siRNA are encapsulated in an alganate‐based hybrid hydrogel (SOG). MSLs strongly down‐regulates the expression of STAT3 in TME, and promotes tumor cell apoptosis and M1 cell polarization. Moreover, the release of LID relieves pain and activates innate immune cells, suggesting the multiple effects of MSL@LID@SOG system combined therapy [96]. Poly (lactic‐co‐glycolic acid) (PLGA)‐polyethylenimine (PEI) nanobubbles (NBs) carrying STAT6 siRNA, not only has biological stability, but also shows excellent ultrasonic imaging ability. Ultrasound‐mediated nanobubble destruction (UMND) enhances the effect of PLGA‐PEINBs‐STAT6 siRNA, promotes M1 polarization, and inhibits the proliferation and EMT of lung cancer cells [97]. Au@PG nanoparticles are gold (Au)‐based NPs featuring a polyaniline‐based glyco structure (PG), and have been shown to activate M1 polarization of macrophages, promote T cell immune response and suppress the tumor development. Au@PG NPs combined with anti‐PD‐1 therapy was found to enhance immune response, increase the level of immune factors, and inhibit tumor progression [98].

## 7 TAMs‐Related NSCLC Clinical Trials

Studies have shown that the TAMs modulate the progression of lung cancer [27, 33, 35]. Currently, clinical trials are investigating new drugs for lung cancer treatment (Table 3).

**TABLE 3:** Clinical trials related to TAMs.

Trial name	TG6050	Mobocertinib/TAK‐788	MRG004A	GB1275	RXDX‐106
Phase	Phase 1	Phase 1/2	Phase 1/2	Phase 1/2	Phase 1
Target population	NSCLC	Carcinoma, NSCLC	Solid tumors	Solid tumors	Solid tumors
Treatment modality	IV	Oral	IV	Oral	Oral
Key outcomes	Changing the proportion of immune cells; increasing the anti‐tumor activity	Good selective distribution; good inhibitory activity	Anti‐tumor; reducing drug resistance	Decreasing TAMs' infiltration; reprogramming M1/M2 phenotype	Activating innate and acquired immunity; inhibiting tumor growth
NCT number	NCT05788926	NCT02716116	NCT04843709	NCT04060342	NCT03454243
Reference	[99]	[100]	[101]	[102]	[103]

TG6050 is an oncolytic vaccinia virus designed to express IL‐12 and an anti‐CTLA‐4 antibody. An analysis of the changes in the TME following TG6050 treatment revealed increased levels of γ‐interferon, CD8+ T cells, and the M1/M2 macrophage ratio. The virus facilitated the delivery and release of IL‐12 and CTLA‐4 directly into the tumor, significantly enhancing the anti‐tumor immune response. These promising preclinical findings support the initiation of a clinical study of TG6050 in metastatic non‐small cell lung cancer (NCT05788926). The study aims to determine the appropriate dosage and administration regimen for TG6050 through single or repeated intravenous infusions while also assessing safety and tolerability to guide further development [99]. Mobocertinib (TAK‐788) is an effective irreversible tyrosine kinase inhibitor (TKI), aiming at inserting mutation into exon 20 of human epidermal growth factor receptor 2 (HER2/ERBB2). Mobocertinib showed good selective distribution in preclinical model, which was superior to other drugs in the experiment, and showed good inhibitory activity. In addition to these effects, the presence of M1 macrophages and CD4+ T cells play a crucial role in inhibiting tumor growth. Furthermore, additional experimental results provide a robust foundation for the clinical investigation of mobocertinib (NCT02716116) and support its continued development. The main purpose of this study is to find out whether TAK‐788 has side effects, and to determine the in vivo process and optimal dosage of TAK‐788 [100]. Tissue factor (TF) is overexpressed in NSCLC KRAS mutation (KRASmut). TF antibody HuSC1‐39 or combined with KRASG12C‐I has anti‐tumor effect. HuSC1‐39 significantly enhanced its phagocytic ability against tumor cells by promoting M1 polarization and inhibiting EMT, thereby increasing the sensitivity of Kras‐G12C inhibitors. A clinical study is currently underway to evaluate the safety, pharmacokinetics, and dosage of MRG004A in the treatment of various diseases (NCT04843709) [101]. CD11b is highly expressed in TAMs, which is an effective therapeutic target. GB1275 is a CD11b regulator, and the related clinical research is under development (NCT04060342). Preclinical studies found that the ratio of M1/M2 TAMs and CD8+ T cells increased in mice treated with GB1275 and CD11b KI [102]. The expression of the receptor tyrosine kinase (RTK) family is associated with cancer progression and drug resistance. RXDX‐106, a small molecule inhibitor targeting TAM RTK, has been shown to activate both innate and acquired immunity. Treatment with RXDX‐106 resulted in tumor growth inhibition, which correlates with the polarization of M1 macrophages and the activation of NK cells in the innate immune response [103]. A clinical study to evaluate the safety and tolerability of RXDX‐106 is under way (NCT03454243), to understand the pharmacokinetics and efficacy of drugs and to achieve better application.

## 8 Discussion

By promoting angiogenesis and EMT, TAMs facilitate tumor invasion, growth, and metastasis. Additionally, they contribute to the development of drug resistance and the establishment of an immunosuppressive microenvironment [26]. In the TME, immunosuppressive factors secreted by TAMs can weaken the viability of immune cells and suppress their killing effect on tumors [104] contributing to immunosuppression and hindering the effectiveness of therapies. From these preclinical studies, we find that TAMs work through different signal pathways, and their influence is extensive. It is meaningless to simply inhibit the secretion of a certain factor or block a certain pathway, so we generally think that the treatment should focus on TAMs itself.

Currently, various strategies are being explored, including removing TAMs, reducing the recruitment of TAMs and influencing their polarization. These are very classic and common means. The removal of TAMs is not completely specific, and the loss of macrophages is inevitable in this process. Theoretically, inhibiting the recruitment of TAMs can reduce the number of M2 macrophages and restore the immune function to some extent, while reprogramming of TAMs can restore the phagocytosis and immune stimulation functions of macrophages along with the functional transformation. However, related experiments have proved that the reactivity and therapeutic effects of these drugs are not in line with expectations. The biological function of TAMs is complex, and the tumor environment is complex, which requires us to explore more accurate targets or seek combined treatment. Future NSCLC treatment strategies should prioritize precise regulation of TAM function [105, 106, 107, 108, 109].

Our focus diverges from these common strategies, as we have summarized the biomarkers and treatment systems associated with TAMs to aid in the treatment and prediction of lung cancer. The expression of these biomarkers is closely linked to immune infiltration and therapeutic response, significantly influencing the recruitment and proportion of TAMs [62, 75, 78]. This insight drives our efforts to develop feasible and beneficial biomarkers for predicting patient prognosis and evaluating potential therapeutic responses in conjunction with other treatment modalities, particularly immunotherapy. The results showed that there are multiple biomarkers, involving ferroptosis, cell cycle regulation, RNA editing, and other biological functions, but it is not clear whether these biomarkers are sensitive, specific, reliable, economical, and repeatable. Individual patient variability is often significant, making it challenging for a single biomarker to reliably predict disease outcomes. Typically, the combined analysis of multiple biomarkers enhances specificity and sensitivity in disease prediction [110]. This observation opens up new experimental avenues: selecting promising biomarkers for combination assessments to evaluate their collective effectiveness.

In addition, the therapeutic system based on TAMs can achieve the precise delivery of drugs or gene interfering molecules, without causing side effects. Effective drug coating and precise release are crucial for successful delivery, posing challenges in selecting appropriate materials for treatment systems. Researchers are actively modifying nanoparticles, such as basic liposomes [94], and exploring biological carriers like extracellular exosomes [79] to combine ray and other signals [88, 91], thereby stimulating drug release and achieving signal amplification. In the future, researchers should improve this treatment system and identify new discoveries and opportunities for patient's treatment.

Several clinical trials have been conducted on NSCLC. The inhibition and deficiency of tyrosine kinases, TFs and CD11b affect the proportion and polarization of TAMs in tumor environment, and slow down the growth and deterioration of tumor. The results of these clinical trials prove that the good therapeutic effect is inseparable from the existence of M1 macrophages, which further affirms the feasibility of TAMs reprogramming strategy. Perhaps, like the current immunotherapy, the strategy of targeting TAMs to enhance immunity still cannot avoid the problem of individual differences, but as an adjuvant therapy, it may have an effect in prolonging the survival of patients.

## Author Contributions

**Tongtong Lv:** data curation (equal); investigation (equal); visualization (equal); writing – original draft (equal). **Rui Fan:** resources (equal); supervision (equal); writing – original draft (equal). **Jiaqi Wu:** formal analysis (equal); supervision (equal). **Haolan Gong:** supervision (equal); visualization (equal). **Xiaoru Gao:** data curation (equal); investigation (equal). **Xin Liu:** investigation (equal). **Yixin Gong:** investigation (equal). **Bo Luo:** investigation (equal). **Yanhua Zhang:** funding acquisition (equal). **Xiaochun Peng:** conceptualization (equal); funding acquisition (equal); project administration (equal); supervision (equal); writing – review and editing (equal). **Gai Liang:** funding acquisition (equal); project administration (equal); supervision (equal).

## Ethics Statement

The authors have nothing to report.

## Consent

The authors have nothing to report.

## Conflicts of Interest

The authors declare no conflicts of interest.

## Data Availability

The data that support the findings of this study are available on request from the corresponding author. The data are not publicly available due to privacy or ethical restrictions.
